# Automatic Detoxification Medicine Delivery by Thermo-Sensitive Poly(ethylene glycol)-Based Nanogels

**DOI:** 10.3390/polym14050892

**Published:** 2022-02-24

**Authors:** Ting Fu, Jing Shen, Yuting Meng, Jun Wang, Siping Wang, Yuhui Zhang, Tongwen Wang, Xufeng Zhang

**Affiliations:** 1 College of Chemistry and Chemical Engineering, Yunnan Normal University, Kunming 650500, China; gracefuting@126.com (T.F.); m18468288406@163.com (Y.M.); 4478@ynnu.edu.cn (J.W.); zyh_12342022@163.com (Y.Z.); wtongw@126.com (T.W.); 2 Institute of Education, China Democratic League (Yunnan), Kunming 650228, China; sipingwang18@163.com

**Keywords:** detoxification medicines delivery, nonlinear poly(ethylene glycol)-based copolymer shell, thermo-sensitive nanogels

## Abstract

During the medication-assisted treatment of drug abuse, side effects and addiction liabilities are commonly observed. Thus, control of the medication dose is very important. According to body temperature abnormalities in drug abusers, a thermo-sensitive nanogel was synthesized as a drug carrier to automatically deliver detoxification medicines. This nanogel was prepared through the synthesis of polystyrene (PS) core microspheres, followed by coverage with a nonlinear poly(ethylene glycol)-based copolymer shell. The PS core microspheres were found to be an ideal hydrophobic core for loading the detoxification medicines effectively. The nonlinear poly(ethylene glycol)-based copolymer shell layer consisted of 2-(2-methoxyethoxy)ethyl methacrylate (MEO_2_MA) and oligo(ethylene glycol) methyl ether methacrylates (*M*_n_ = 300 g mol^−^^1^, MEO_5_MA). The monomer feeding molar ratio *n*(MEO_2_MA)/*n*(MEO_5_MA) of 1:3 enabled PS@P(MEO_2_MA-*co*-MEO_5_MA) nanogels to exhibit a distinguished colloidal stability and an adjustable volume phase transition temperature which is within the drug addicts’ abnormally fluctuating temperature range. Importantly, it was found that the obtained PS@P(MEO_2_MA-*co*-MEO_5_MA) nanogels displayed good biocompatibility with rat aortic endothelial cells in the given concentration range. The nanogels also exhibited a satisfactory loading efficiency and thermo-sensitive/sustained release characteristics for three detoxification medicines: sinomenine, diltiazem and chlorpromazine.

## 1. Introduction

Drug abuse is one of the most serious social problems around the world today [1]. Yunnan province, the hometown of one of the authors, is located in the southwest of China. The unique climate and political environment have made this district one of the largest opium planting areas in China. Consequently, increasing attention has been paid to the issue of control, treatment and rehabilitation of drug addiction. Drug addicts often need to take certain detoxification medicines [2,3]. However, side effects and addiction liabilities are commonly observed in the period of medication-assisted treatment [4,5]. At this point, how the dose of the medications is controlled is critical. Generally, fluctuating and higher body temperatures have been observed in persons with addictions. For example, Lu et al. observed that in 1278 amphetamine or dope abusers, the average body temperature was determined to be 37.3 ± 3.2 °C. Meanwhile, an average body temperature of 36.5 ± 0.7 °C was found in the control group [6]. Obviously, compared with normal people, the body temperature of drug abusers is higher and more fluctuated. In order to improve the utilization rate and reduce the side effects of detoxification medicines, a drug delivery carrier would be a wonderful option if it could automatically release detoxification medicines along with body temperature abnormalities.

Environmentally sensitive nanogels have been explored as drug delivery carriers for biological applications [7,8]. Among a variety of nanogels, core-shell structure nanogels can provide an enhanced efficacy for loading a medicine with their tunable hydrophobic cores, and achieve the controlled release of a medicine through their smart shell components [9]. Recently, sensitive shell components have been thoroughly explored for drug delivery applications. Environmental stimuli include changes in pH, temperature, light, concentration of glucose and so on [10,11,12,13]. Thermo-sensitive poly(N-isopropylacrylamide) (PNIPAM)-based nanogels are one of these previous study objects [14,15]. However, it was found that the application of these PNIPAM-based nanogels is limited because of their inherent biocompatibility problem [16]. To better adapt the biocompatibility of drug delivery systems, a new thermo-sensitive polymer containing short-side-chain oligo(ethylene glycol) units was developed [17]. It was reported that controlling the components of the oligo(ethylene glycol) side-chain units can adjust the lower critical solution temperature (LCST) of this polymer. For example, a tunable LCST was achieved with the different comonomer ratios of 2-(2-methoxyethoxy)ethyl methacrylate (MEO_2_MA) and oligo (ethylene glycol) methyl ether methacrylate (*M*_n_ = 475 g mol^−1^, MEO_9_MA) [18,19,20]. According to Ishizone et al., the oligo(ethylene glycol) side-chain length determined the LCST of the poly[oligo (ethylene glycol) alkyl ether methacrylates] [21,22]. Recently, this nonlinear poly(ethylene glycol)-based copolymer was used to loading/release active bio(macro)molecules [23]. The core-shell structure nanogels with a tunable polymer core and thermo-responsive short oligo(ethylene glycol) side-chain shell have exhibited obvious advantages for drug loading/release and chemotherapy [24,25]. In these core-shell structure nanogels, a hydrophobic polymer core was usually used to load hydrophobic medicines, and a hydrophilic shell with a short oligo(ethylene glycol) side-chain copolymer provided a thermo-sensitive function to effectively release the medicines [26]. We believe that the efficient loading of high-detoxification medicines and smart stimuli release can be achieved if a proper hydrophobic polymer core is combined with a thermo-sensitive nonlinear poly(ethylene glycol)-based copolymer shell.

**Scheme 1:**
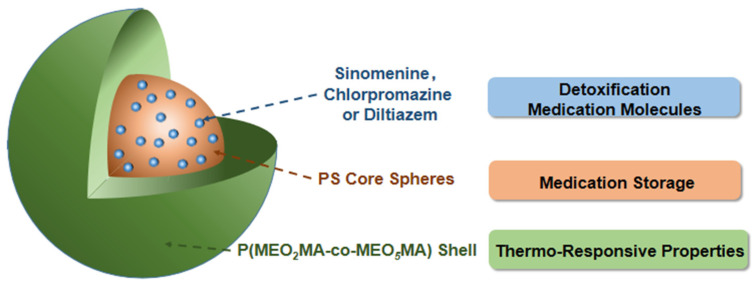
Schematic illustration of PS@P(MEO_2_MA-*co*-MEO_5_MA) core-shell nanogel loaded with detoxification medicines.

To demonstrate this concept, we report a preparation of core-shell structured nanogels with a polystyrene (PS) microsphere as the core and PEG-copolymers containing short-side-chain oligo(ethylene glycol) units as the shell to integrate the high-detoxification medicine loading yields and thermo-sensitive release. As shown in Scheme 1, the core is composed of a hydrophobic PS microsphere, which could provide a hydrophobic network structure to load detoxification medicines. The shell layer consists of a hydrophilic copolymer of 2-(2-methoxyethoxy)ethyl methacrylate (MEO_2_MA) and oligo(ethylene glycol) methyl ether methacrylates (MEO_5_MA), which is expected to offer a tunable thermo-sensitive control in the loading/release of detoxification medicines. The effect of the initial shell monomer feeding amount on the thermo-sensitive characteristic of the synthesized PS@P(MEO_2_MA-*co*-MEO_5_MA) nanogels was investigated in detail. The biocompatibility of the synthesized nanogels was also tested. Moreover, three detoxification medicines (sinomenine, diltiazem and chlorpromazine) were selected as model drugs to investigate the loading/release behaviors of the synthesized core-shell nanogels, along with the environmental temperature changes.

This class of PS/P(MEO_2_MA-*co*-MEO_5_MA) nanogels could provide a strong hydrophobic PS core to load more detoxification agents (sinomenine, diltiazem and chlorpromazine). At the same time, it would offer the automatic release of detoxification medicines along with body temperature abnormalities of drug abusers by means of the thermosensitive shell composed of P(MEO_2_MA-co-MEO_5_MA). This carrier will reduce the frequency of drug administration and minimize the side effects. Thus, it will bring great convenience to patients and definitely alleviate their pain.

## 2. Materials and Methods

### 2.1. Materials

2-(2-methoxyethoxy)ethyl methacrylate (MEO_2_MA, 95%), oligo(ethylene glycol)methyl ether methacrylate (*M*_n_ = 300 g mol^−1^, MEO_5_MA), poly(ethylene glycol) dimethacrylate (PEGDMA, *M*_n_ ≈ 550 g mol^−1^), divinylbenzene (DVB) and 2,2′-azobis(2-methylpropionamidine) dihydrochloride (AAPH) were purchased from Sigma-Aldrich. 1-methylimidazole and 1-hexadecyl chloride were purchased from Acrös Organics. Long-chain ionic liquid 1-hexadecyl-3-methylimidazolium chloride (IL_16_) was synthesized according to the original report [27]. Styrene (St) and sodium dodecylbenzene sulfonate (SDBS) were purchased from Shanghai Titan Technology Co., Shanghai, China. Sinomenine (SIN), diltiazem (DIZ) and Chlorpromazine (CPZ) were purchased from Meilun Biological Products Company, Dalian, China. All reagents were used as received without further purification.

### 2.2. Preparation of Polystyrene (PS) Core Microspheres

Monodisperse PS microspheres were synthesized through emulsion polymerization [28], but emulsifier sodium dodecyl sulfate (SDS) was replaced by sodium dodecylbenzene sulfonate (SDBS). Typically, 4.5 mL of St was decanted into a separating funnel, washed four times with 20 mL of NaOH solution (0.1 mol L^−1^), and then washed four times with deionized water in turn. 70 mL of ethanol-water mixed solution with an ethanol/water volume ratio of 2:5 was added to a round-bottom burning screen, and then 0.0050 g SDBS was added. After stirring, the washed St and 8 μL of DVB were added to the ethanol-water mixed solution. The mixture was heated to 70 °C under a N_2_ purge. After 30 min, 0.10 g initiator potassium persulfate (K_2_S_2_O_8_) was poured into the solution. The polymerization was carried out for 8 h. The synthesized PS core microspheres were purified by centrifugation (2500 rpm min^−1^, 2 h)/redispersion method twice, followed by drying at room temperature.

### 2.3. Preparation of PS@P(MEO_2_MA-co-MEO_5_MA) Nanogels

A 100 mL water suspension of 0.0235 g of dried PS microspheres was stirred under ultrasound with 10 mL of 0.019 g mL^−1^ IL_16_ solution as a dispersant. The dispersion process was allowed to process for 8 h. The PS microsphere cores were used as nuclei to prepare the PS@P(MEO_2_MA-*co*-MEO_5_MA) nanogels by precipitation polymerization. A certain amount of shell precursor (MEO_2_MA and MEO_5_MA) with a feeding molar ratio of 1:3 and PEGDMA crosslinker (see Table 1 for specific dosage) were added into the PS dispersion. After stirring, the mixture was heated to 70 °C under an N_2_ purge for 1 h, and then the AAPH initiator was added to initiate the polymerization. This reaction was carried out for 5 h. The resulting PS@P(MEO_2_MA-*co*-MEO_5_MA) nanogels were purified by centrifugation (100,000 r min^−1^), decantation and redispersion in deionized water three times. Then, Spectra/Por molecularporous membrane tubing with a molecular weight cutoff of 12,000–14,000 was used for dialysis at room temperature for 3 days.

### 2.4. Cell Viability Evaluation of Nanogels

In a 96-well plate (Costar, approximately 4 × 10^3^ cells per well), rat aortic endothelial cells (RAECs) were cultured with DMEM medium containing 10% FBS and exposed to PS@P(MEO_2_MA-*co*-MEO_5_MA) nanogel dispersions with different concentrations for 24 h. The measurement of the viability of the RAECs was carried out by MTT assay [29]. The specific steps were as follows: 20 μL of MTT (5 mg mL^−1^, dissolved in phosphate buffer solution, pH = 7.4) was added to each well. After incubation for 4 h, the medium was aspirated, and the intracellular formazan crystals were extracted into 150 mL of DMSO. After gentle mixing for 10 min, the absorbance of the cell lysate at 490 nm was measured. The cell viability was expressed as the percentage of the sample absorbance over the control absorbance [30].

### 2.5. Drug Loading and Release

Three detoxification medicines, sinomenine (SIN), diltiazem (DIZ) and chlorpromazine (CPZ), were loaded into the PS@P(MEO_2_MA-*co*-MEO_5_MA)-2 nanogels by complexation method. The following steps take SIN as an example. Firstly, 5 mL of the PS@P(MEO_2_MA-*co*-MEO_5_MA)-2 nanogel dispersion was stirred in an ice-water bath for 30 min to allow the nanogels to fully swell. Subsequently, 5 mL of 2 mg mL^−1^ fresh SIN solution was added to the nanogel dispersion drop by drop. After stirring for 1 day, the dispersion was centrifuged at 10,000 rpm min^−1^ for 15 min. The upper solution was collected and the precipitate was redispersed in 5 mL of buffer solution (pH = 7.4). The sample was further purified by repeated centrifugation/washing steps at least three times. All the upper clear solution from each centrifugation process was combined. The concentration of free SIN in the upper clear solution was determined by UV-vis spectrometry at 263 nm, based on the linear calibration curve (R^2^ > 0.99) which was measured using the SIN solutions with known concentrations under the same conditions. The amount of SIN loaded into the PS@P(MEO_2_MA-*co*-MEO_5_MA)-2 nanogels was calculated from the decrease in SIN concentration. The encapsulation efficiency of the PS@P(MEO_2_MA-*co*-MEO_5_MA)-2 nanogels was calculated as follows:

$$\mathrm{SIN}\;\mathrm{encapsulation}\;\mathrm{efficiency}\;\left(\%\right)=\frac{W\left(\mathrm{feeding}\;\mathrm{SIN}\right)-W(\mathrm{free}\;\mathrm{SIN})}{W(\mathrm{feeding}\;\mathrm{SIN})}\times 100\%$$

The in vitro release test of the SIN-loaded PS@P(MEO_2_MA-*co*-MEO_5_MA)-2 nanogels was evaluated by the dialysis method. First, the purified SIN-loaded PS@P(MEO_2_MA-*co*-MEO_5_MA)-2 solid was redispersed in deionized water (5 mL). Then, 1 mL of the SIN-loaded PS@P(MEO_2_MA-*co*-MEO_5_MA)-2 dispersion was transferred into a Spectra/Por molecularporous membrane tube (molecular weight cutoff of 12,000–14,000) for dialysis. Subsequently, the dialysis bag was immersed into 50 mL of phosphate buffer solution (0.005 M) at both 22 °C and 38 °C. At defined time intervals, the SIN released outside of the dialysis bag was sampled and assayed at 263 nm using UV-vis absorption. The total percentage of SIN released outside the dialysis bag was expressed as a cumulative release. Blank release experiments, which contained a free SIN solution with the same amount of SIN loaded into the PS@P(MEO_2_MA-*co*-MEO_5_MA) nanogels, were carried out under the same conditions at 38 °C.

The same loading and release methods were applied for DIZ and CPZ, except the tested maximum adsorption wavelengths were 236 and 306 nm for DIZ and CPZ, respectively.

### 2.6. Characterization

A field emission scanning electron microscopy (FEI Nova NanoSEM 450, Singapore, Singapore) was employed to observe the morphology of the PS core microspheres. A JEM-2100 electron microscope with an acceleration voltage of 200 kV was used to take transmission electron microscopy (TEM) images of the nanogels. In the TEM measurements, approximately 1 mL of the sample suspension was air-dried on a carbon-coated copper grid (300 meshes). The sizes of the nanogels were measured by a submicron laser particle size analyzer (Brookhaven, BI-90 Plus) at different temperatures. Absorption spectra of the solutions were collected by using a UV-vis spectrophotometer (SHIMADZU UV-1780, Kyoto, Japan).

## 3. Results and Discussion

### 3.1. Morphology of PS Core and PS@(MEO_2_MA-co-MEO_5_MA) Nanogels

**Figure 1:**
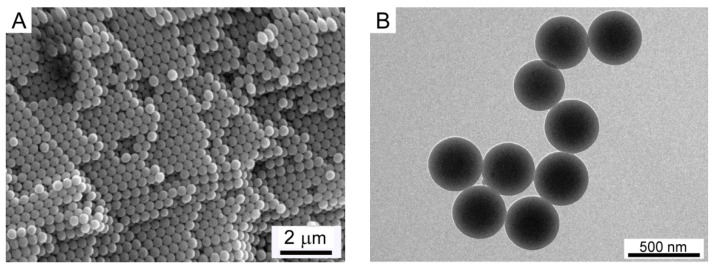

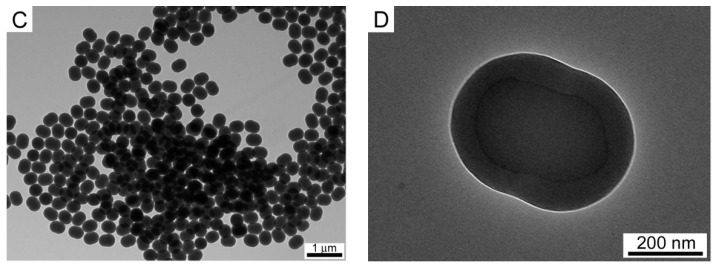
(**A**) SEM and (**B**) TEM images of the PS microspheres. (**C**) TEM image of PS@P(MEO_2_MA-*co*-MEO_5_MA) nanogel particles. (**D**) Single particle TEM image of PS@P(MEO_2_MA-*co*-MEO_5_MA).

Monodisperse PS microspheres were synthesized by emulsion polymerization [28], but the emulsifier SDS was replaced by sodium dodecylbenzene sulfonate (SDBS). The SEM image (Figure 1A) clearly demonstrates spherical PS particles with a uniform and spherical morphology. From the TEM image (Figure 1B), the diameter of the monodisperse PS microspheres was determined to be 350 ± 5 nm. Figure 1C shows the TEM image of the PS@P(MEO_2_MA-*co*-MEO_5_MA) core-shell nanogels. The particles exhibited a uniform size and elliptic morphology, which indicated that when the spherical PS core microspheres were coated with a nonlinear poly(ethylene glycol)-based copolymer, the morphology of the particles could change from spherical PS core to ellipsoidal PS@P(MEO_2_MA-*co*-MEO_5_MA) core-shell nanogel particles. Figure 1D further shows a high-resolution TEM image of a single particle of the PS@P(MEO_2_MA-*co*-MEO_5_MA) nanogel. From this image, a clear core-shell boundary can be observed in the particle. The ellipsoidal particle, with a long axis of about 480 ± 5 nm and short axis of 376 ± 5 nm, is significantly larger than the PS core (350 ± 5 nm). The inner core of the ellipsoidal particle was estimated to be about 373 ± 5 nm along the long axis and 235 ± 5 nm along the short axis, indicating that the PS core was squeezed by the P(MEO_2_MA-*co*-MEO_5_MA) shell. Moreover, the wall thickness was approximately 72 ± 3 nm, which is attributed to the thickness of the shell.

### 3.2. Thermo-Sensitive Behavior of PS@(MEO_2_MA-co-MEO_5_MA) Nanogels

**Figure 2:**
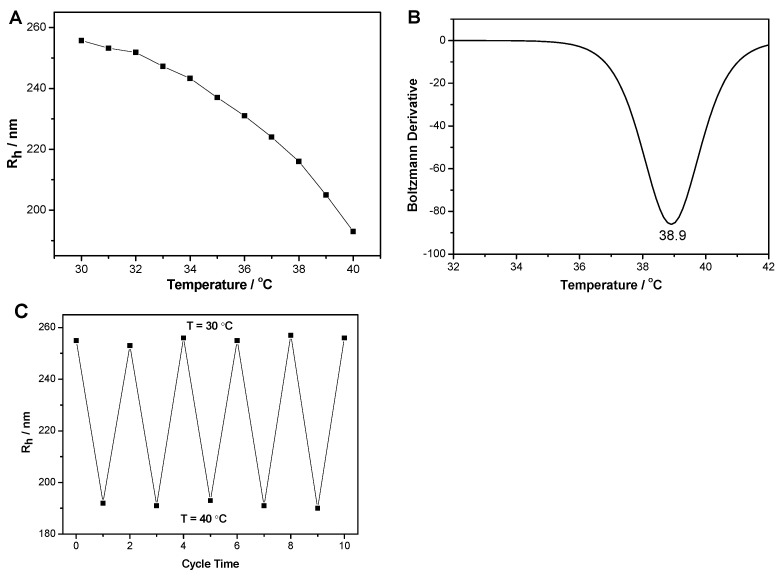
(**A**) Temperature-dependent average radius (*R*_h_) value of PS@P(MEO_2_MA-*co*-MEO_5_MA) nanogels, (**B**) its corresponding Boltzmann fitting-differential curve, (**C**) Reversible *R*_h_ value change of the hybrid nanogels after five cycles of repeated heating (40 °C) and cooling (30 °C).

The PS@P(MEO_2_MA-*co*-MEO_5_MA) nanogels were synthesized by feeding MEO_2_MA (2.5 mmol) and MEO_5_MA (7.5 mmol) in 100 mL of 0.227 g L^−1^ PS dispersion. Figure 2A shows the temperature-dependent average radius (*R*_h_) of the nanogel particles. It is clear that the *R*_h_ of the nanogel decreases from 255 nm to 193 nm when the temperature increases from 30 °C to 40 °C. That is, the temperature of the dispersion medium can significantly influence the size of the PS@P(MEO_2_MA-*co*-MEO_5_MA) nanogels. The change in volume can be attributed to a thermo-sensitive nonlinear PEG-based gel shell, because the change in volume of the pure PS microspheres was not observed at the corresponding temperature range. Referring to [31], and using the method of corresponding Boltzmann fitting-differential curve, the volume phase transition temperature (VPTT) of the nanogels was determined to be 38.9 °C (Figure 2B). This is within the fluctuating temperature scale of drug addicts. Figure 2C shows the *R*_h_ value change of the PS@P(MEO_2_MA-*co*-MEO_5_MA) nanogels experiencing five cycles of heating and cooling adjustment. The particle size was fully reproducible after repeated heating and cooling, indicating the particles can shrink and swell reversibly. This is due to the reversible thermo-responsive volume phase transition of the nonlinear PEG-based gel shell. The reversible change of the particles’ size is critical for the nanogels to automatically release drugs upon environmental stimuli.

**Figure 3:**
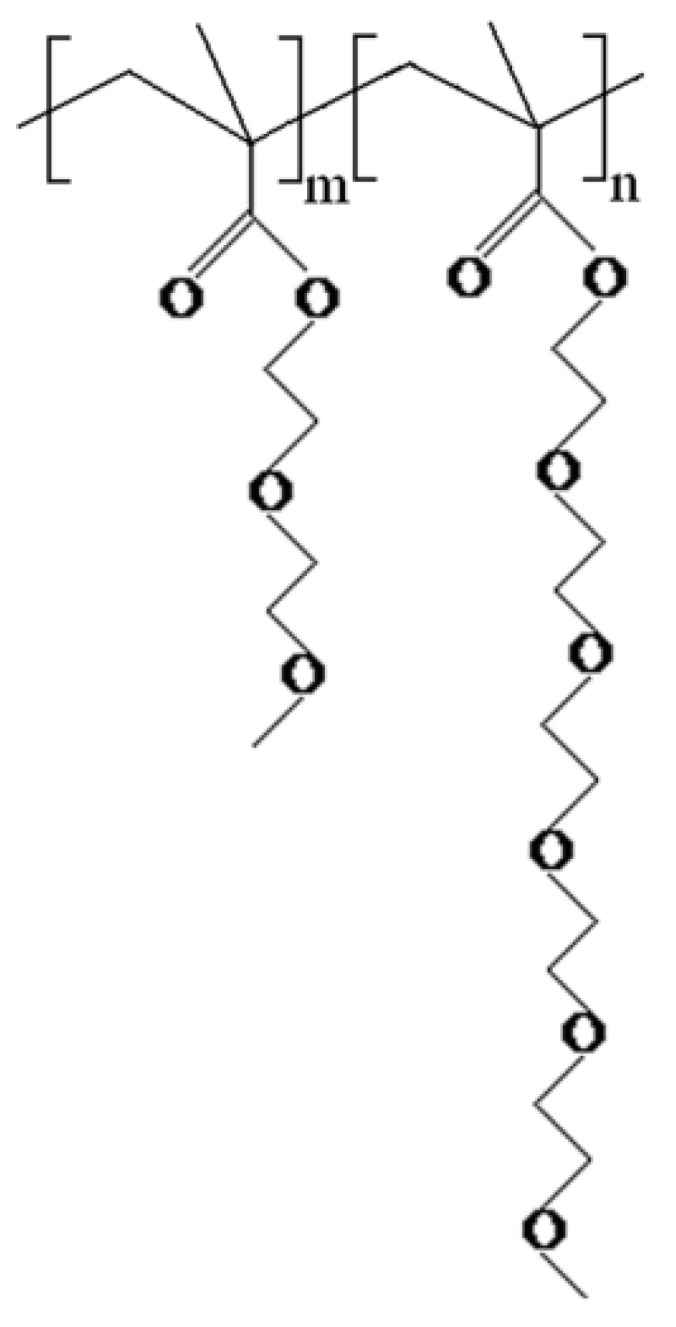
Structure diagram of P(MEO_2_MA-*co*-MEO_5_MA) copolymer.

Why do the volumes of the nanogels change along with the ambient temperature? Let us take a look at the structure of the nonlinear poly(ethylene glycol)-based P(MEO_2_MA-*co*-MEO_5_MA) shell copolymer in Figure 3. For example, when the temperature of the dispersion medium is lower than 30 °C, the temperature of shell component P(MEO_2_MA-*co*-MEO_5_MA) is lower than its VPTT. A hydrogen bond may be formed between the oxygen atoms of the short oligo(ethylene glycol) side chain and the hydrogen atoms of the aqueous medium. This hydrogen bond impels water molecules to enter into the nanogel network. This finally leads to volume swelling of the whole nanogel. On the contrary, when the temperature of the dispersion medium is higher than its VPTT, the hydrogen bond is broken. Water molecules in the nanogel network may be squeezed out, resulting in the contraction of the entire nanogel.

**Figure 4:**
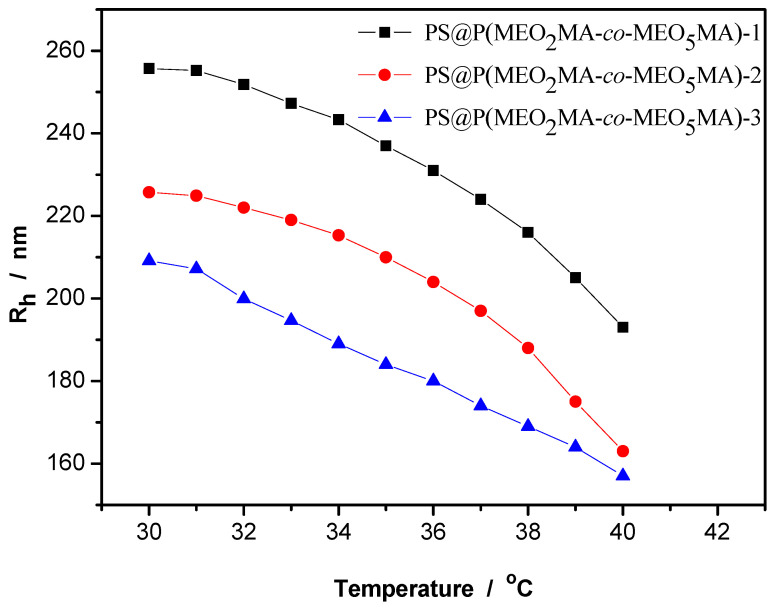
Temperature-dependent average radius (*R*_h_) value of PS@P(MEO_2_MA-*co*-MEO_5_MA) nanogels prepared with the same *n*(MEO_2_MA)/*n*(MEO_5_MA) feeding molar ratio of 1:3 but different initial monomer (MEO_2_MA and MEO_5_MA) amounts of shell in 100 mL of 0.227 g L^−1^ PS dispersion.

Figure 4 further shows the temperature-dependent average radius (*R*_h_) of the PS@P(MEO_2_MA-*co*-MEO_5_MA)-x (x = 1, 2, 3) nanogels synthesized with the same *n*(MEO_2_MA)/*n*(MEO_5_MA) feeding molar ratio of 1:3 but with different initial feeding amounts of MEO_2_MA and MEO_5_MA (see details in Table 1). As can be seen from Figure 4, three samples still keep a tendency of volume contraction according to an increase in ambient temperature from 30 to 40 °C, which shows obvious temperature sensitive characteristics. Another fact is that the sizes of the nanogels become slightly smaller when the number of two-shell monomers increases at the corresponding temperature. This may be due to an increase in the amounts of two-shell monomers, which leads to an increase in the outer shell layer thickness of the nanogels, further squeezing the PS cores. Figure S1 shows the corresponding Boltzmann fitting-differential curves of three samples. The VPTT values of the three samples were determined to be 38.9 °C, 38.5 °C and 32.5 °C for PS@P(MEO_2_MA-*co*-MEO_5_MA)-1, PS@P(MEO_2_MA-*co*-MEO_5_MA)-2 and PS@P(MEO_2_MA-co-MEO_5_MA)-3, respectively. From the perspective of the temperature fluctuation range of 37.24–40.50 °C in drug addicts in the throes of an addiction, we chose PS@P(MEO_2_MA-*co*-MEO_5_MA)-2 for further experimental research.

### 3.3. Biocompatibility Assay

**Figure 5:**
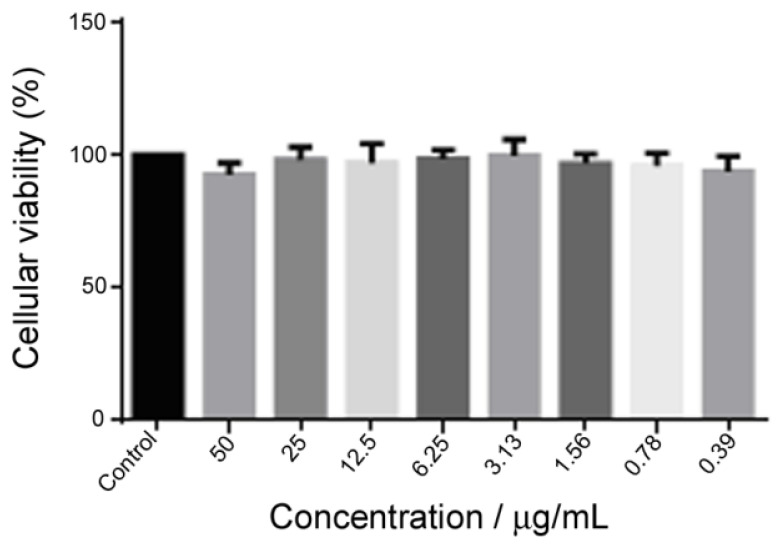
In vitro RAECS viability following treatments with PS@P(MEO_2_MA-*co*-MEO_5_MA) nanogels at different concentrations.

An MTT assay was employed to evaluate the viability of the rat aortic endothelial cells (RAECs) in the presence of the PS@P(MEO_2_MA-*co*-MEO_5_MA) nanogels. Figure 5 displays the survival rate of the RAECs after being exposed to the aqueous PS@P(MEO_2_MA-*co*-MEO_5_MA) nanogel dispersion with concentrations of 50.0, 25.0, 12.5, 6.25, 3.12, 1.56, 0.78 and 0.39 μg mL^−1^ for 24 h. These results indicate that more than 90% of RAECs can survive in concentrations of up to 50 μg mL^−1^, exhibiting an excellent in vitro biocompatibility in the given concentration range.

### 3.4. Drug Loading and In Vitro Thermo-Sensitive Regulated Drug Release

#### 3.4.1. Drug Loading Efficiency

Sinomenine, diltiazem and chlorpromazine were chosen as model detoxification medicines. Figure S2 shows the chemical structures of the three medicines. PS@P(MEO_2_MA-*co*-MEO_5_MA)-2 nanogels were selected as a carrier to assess drug loading efficiency through the usual physical adsorption method.

Sinomenine (SIN) (Figure S2A) is a monomer of an effective alkaloid, extracted from the roots of sinomenium acutum (a traditional Chinese medicine). Its structure is similar to morphine and is a histamine releasing agent. SIN has anti-inflammatory, antioxidant, analgesic and immunosuppressive effects [32]. Further studies have indicated that SIN can alleviate the symptoms of morphine dependence in rats [33]. In order to improve the clinical efficacy and drug use efficiency of SIN, the SIN must be loaded into a carrier. For example, Chen et al. [34] loaded SIN into the polymer of β-cyclodextrin and chitosan, and modified the molecular structure of the polymer by adjusting the monomer concentration and the amount of crosslinker. It was found that the encapsulation efficiency of the SIN in the polymer could be significantly improved.

Diltiazem (DIZ) (Figure S2B) is a known antagonist of calcium ion, usually used to treat cardiovascular diseases. It was reported that drug addiction can be inhibited by DIZ to some degree [35]. For instance, research shows that DIZ can significantly inhibit the withdrawal reaction and decrease the level of plasma monoamine transmitters in morphine-withdrawing mice [36]. From the point of the inhibitory effect of DIZ on the drug addiction, an effective carrier for DIZ is required. Xu et al. [37] reported that a controlled drug release system of poly(2-hydroxyethyl methacrylate (PHEMA) microgels was effective. The DIZ-loaded PHEMA microgel membrane exhibited a slow release phenomenon within 12 h.

Chlorpromazine (CPZ) (Figure S2C) is a dopamine-receptor blocker in the central nervous system. CPZ usually has the functions of sedation, tranquility, hypnosis and anesthesia, etc. At the same time, CPZ can block the dopamine receptor in the hypothalamus, disturb endocrine function and accelerate the exudation of pituitary auxin, thus restraining the release of prolactin indirectly. Consequently, CPZ is usually applied in heroin addiction treatment, which can reduce the emergency of the body and finally achieve the goal of detoxification. Recently, some research work has also been devoted to investigating the efficacy, stability and safety of CPZ. For instance, Jiang et al. [38] found that the yeast cell wall can be used as capsule material to prepare microcapsules. This CPZ-loaded microcapsule can increase the slow-release and stability of CPZ. Mohammed et al. [39] used a copolymer of poly(lactic acid-*co*-glycolic acid) (PLGA) as a carrier to load CPZ, and found that higher entrapment efficiency and drug loading could be obtained by adjusting the concentration of CPZ, PLGA, polyvinyl alcohol and so on.

The encapsulation efficiency of PS@P(MEO_2_MA-*co*-MEO_5_MA)-2 nanogels was determined to be 74.33%, 77.6% and 70.70% for SIN, DIZ and CPZ, respectively. Moreover, the encapsulation efficiency of pure PS core was determined to be 78.5%, 79.03% and 80.4%, for SIN, DIZ and CPZ, respectively, which is close to that of PS@P(MEO_2_MA-*co*-MEO_5_MA)-2 nanogels. These results suggest that the loading of the three detoxification medicines is mainly borne by the PS core in PS@P(MEO_2_MA-*co*-MEO_5_MA)-2 nanogels. The three detoxification medication molecules passed through the P(MEO_2_MA-*co*-MEO_5_MA)-2 shell into the PS core. This is because heterocyclic aromatic groups of detoxification medication molecules and hydrophobic network chain in the PS core may form intermolecular complexes by means of a hydrophobic interaction.

#### 3.4.2. In Vitro Release Studies

**Figure 6:**
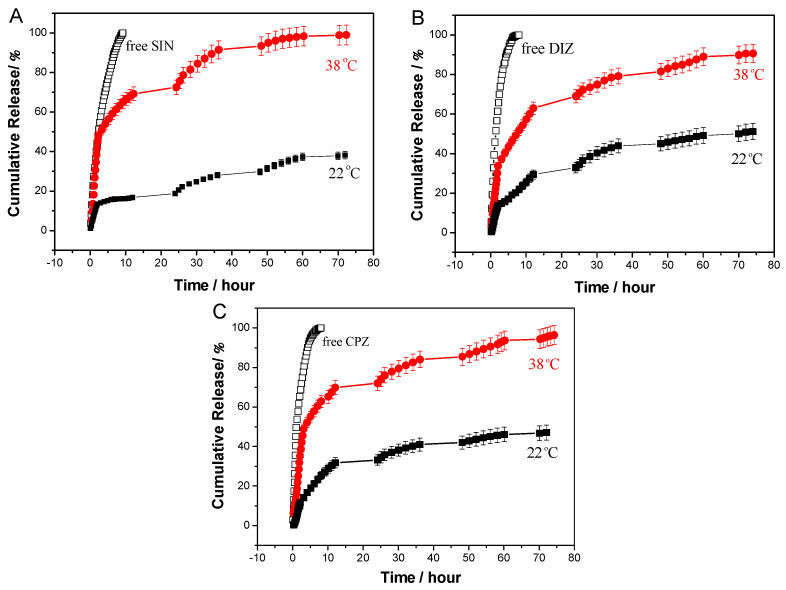
Cumulative release of (**A**) SIN-, (**B**) DIZ- and (**C**) CPZ-loaded PS@P(MEO_2_MA-*co*-MEO_5_MA)-2 in buffer solution at both 22 °C (■) and 38 °C (●) under different times. In the blank (□), a diluted solution of free detoxification medicines with the same amount of medicines loaded in PS@P(MEO_2_MA-*co*-MEO_5_MA)-2 was performed at 38 °C.

The cumulative release profiles of SIN, DIZ and CPZ from the PS@P(MEO_2_MA-*co*-MEO_5_MA)-2 nanogels are shown in Figure 6. The in vitro release studies were carried out in a buffer solution to avoid the decomposition of SIN, DIZ and CPZ in water over a long period of time. As can be seen in the curves (□) of Figure 6A–C, three blank release experiments of free SIN, DIZ and CPZ solutions with the equivalent amount of SIN, DIZ and CPZ loaded in the PS@P(MEO_2_MA-*co*-MEO_5_MA)-2 nanogels, respectively, were carried out under the same conditions at 38 °C. These curves of blank release exhibited a very rapid release in a few minutes, which shows that the role of dialysis membrane is ignorable in the release process. It is clear that if we compare these release curves between the 22 °C and 38 °C in Figure 6A–C, the distinct differences can be observed. With the prolongation of release time, the cumulative release rates of the three samples at 38 °C are higher than that at 22 °C, indicating that this PS@P(MEO_2_MA-*co*-MEO_5_MA)-2 nanogel has significant thermo-sensitive release characteristics. The results can be attributed to the thermo-sensitive shell. When the ambient temperature increases, the P(MEO_2_MA-*co*-MEO_5_MA) shrinks. This shrinkage further compresses the PS core, squeezing out the drug molecules. Furthermore, a sustained-release phenomenon can also be observed in Figure 6A–C. For example, the cumulative release rates of the PS@P(MEO_2_MA-*co*-MEO_5_MA)-2 nanogels reached 98.0%, 90.3% and 95.0% for SIN, DIZ and CPZ, respectively, at 38 °C in 70 h, indicating that the SIN-, DIZ- and CPZ-loaded PS@P(MEO_2_MA-*co*-MEO_5_MA)-2 nanogels can provide a sustained release of detoxification medicines. This is probably due to the slow diffusion of the three detoxification medication molecules from the PS cores of nanogels to the P(MEO_2_MA-*co*-MEO_5_MA) shell layer, and then slowly spreading from the shell layer to the buffer solution. This sustained-release effect is very important for the utilization of drugs, especially for the stability and safety of detoxification medicines. One foreseeable prospect is that the drug-loaded nanogels can be used as an oral drug. When the nanogels are made into an oral liquid, it may enter the body through adsorption in the stomach or intestines. When drug addicts are addicted to drugs, their body temperature rises, and the drug-loaded nanogels can automatically release the drug that has been loaded. It may be a sustained release process. The automatic release of the drug can alleviate the dependence of the human body on drugs.

## 4. Conclusions

Well-defined core-shell structured PS@P(MEO_2_MA-*co*-MEO_5_MA) nanogels have been successfully synthesized through the preparation of a hydrophobic PS core, together with the immobilization of a thermo-sensitive nonlinear poly(ethylene glycol)-based copolymer P(MEO_2_MA-*co*-MEO_5_MA) shell on PS core microspheres. The inner hydrophobic PS core microspheres can provide high loading capabilities for hydrophobic sinomenine, diltiazem and chlorpromazine. The thermo-sensitive outer nonlinear poly(ethylene glycol)-based copolymer P(MEO_2_MA-*co*-MEO_5_MA) shell consisted of an initial monomer feeding molar ratio *n*(MEO_2_MA)/*n*(MEO_5_MA) of 1:3 and the matching feeding amounts, e.g., MEO_2_MA (0.25~0.50 mmol) and MEO_5_MA (0.75~1.5 mmol) in 100 mL of 0.227 g L^−1^ PS dispersion. This optimum designed shell layer can provide good biocompatibility within a certain concentration range (<50 μg mL^−1^), excellent stability in aqueous media, and thermally regulated drug release in the temperature fluctuations during drug addiction. Moreover, the obtained sinomenine-, diltiazem- and chlorpromazine-loaded PS@P(MEO_2_MA-*co*-MEO_5_MA) nanogels showed obvious in vitro thermo-sensitive and sustained-release characteristics up to 70 h. The PS@P(MEO_2_MA-*co*-MEO_5_MA) nanogels designed by the rational integration of a drug-loaded PS core and thermo-sensitive nonlinear poly(ethylene glycol)-based copolymer shell should offer broad opportunities to effectively improve the utilization rate and reduce the side effects of detoxification medicines.

## Figures and Tables

**Table 1:** Recipes used to prepare PS@P(MEO_2_MA-*co*-MEO_5_MA) nanogels with the same *n*(MEO_2_MA)/*n*(MEO_5_MA) feeding molar ratio of 1:3 but different initial feeding amounts of MEO_2_MA and MEO_5_MA.

Sample	MEO_2_MA (mmoL)	MEO_5_MA (mmoL)	AAPH (mmoL)	PEGDMA (mmoL)
PS@P(MEO_2_MA-*co*-MEO_5_MA)-1	0.25	0.75	0.02	0.75 × 10^−2^
PS@P(MEO_2_MA-*co*-MEO_5_MA)-2	0.50	1.50	0.02	1.51 × 10^−2^
PS@P(MEO_2_MA-*co*-MEO_5_MA)-3	0.75	2.25	0.02	2.06 × 10^−2^

## Data Availability

The data presented in this study are available on request from the corresponding author.

## Supplementary Materials

The following supporting information can be downloaded at: https://www.mdpi.com/article/10.3390/polym14050892/s1, Figure S1: Boltzmann fitting-differential curves dependent on temperature-*R*_h_ values of (A) PS@P(MEO_2_MA-co-MEO_5_MA)-1, (B) PS@P(MEO_2_MA-*co*-MEO_5_MA)-2, and (C) PS@P(MEO_2_MA-*co*-MEO_5_MA)-3 nanogels prepared with the same *n*(MEO_2_MA)/*n*(MEO_5_MA) feeding molar ratio of 1:3 but different initial monomer (MEO_2_MA and MEO_5_MA) amounts of shell in 100 mL of 0.227 g L^−1^ PS dispersion; Figure S2. Chemical structures of (A) sinomenine, (B) diltiazem and (C) chlorpromazine.
