# Supplementary material for: Automatic Detoxification Medicine Delivery by Thermo-Sensitive Poly(ethylene glycol)-Based Nanogels
Source: Polymers (Basel). 2022 Feb 24;14(5):892. doi: 10.3390/polym14050892 (PMC8912541; doi:10.3390/polym14050892)
Supplement: Supplementary file 1 [file polymers-14-00892-s001.zip › polymers-1572623-supplementary.pdf]

Supporting Information

Automatic Detoxification Medicine Delivery by Thermo-Sensitive Poly(ethylene glycol)-Based Nanogels

Ting Fu [1], Jing Shen [1,*], Yuting Meng [1], Jun Wang [1], Siping Wang [2], Yuhui Zhang [1], Tongwen Wang [1] and Xufeng Zhang [1,*]

[1] College of Chemistry and Chemical Engineering, Yunnan Normal University, Kunming 650500, China; gracefuting@126.com (T.F.); m18468288406@163.com (Y.M.); 4478@ynnu.edu.cn (J.W.); zyh_12342022@163.com (Y.Z.); wtongw@126.com (T.W.)

[2] Institute of Education, China Democratic League (Yunnan), Kunming 650228, China; sipingwang18@163.com

* Correspondence: shenjing@ynnu.edu.cn (J.S.); xfzhang@ynnu.edu.cn (X.Z.)

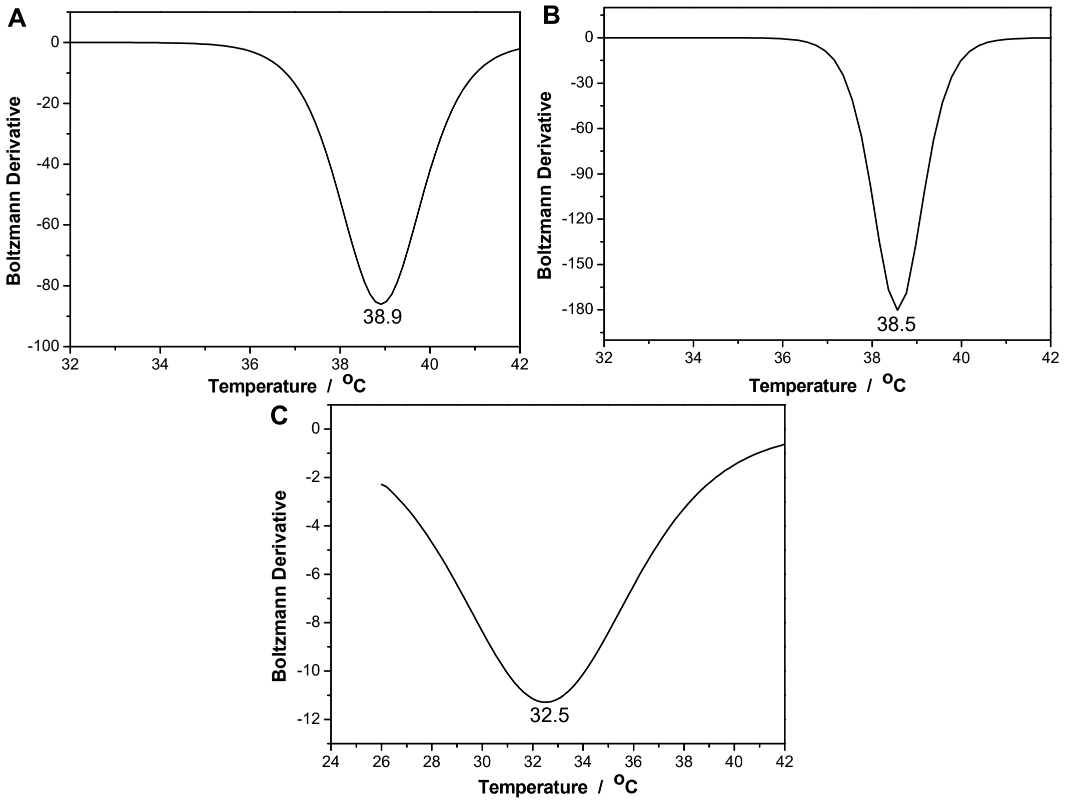

Figure S1. Boltzmann fitting-differential curves dependented on temperature-R_h values of (A) PS@P(MEO$_2$MA-*co*-MEO$_5$MA)-1, (B) PS@P(MEO$_2$MA-*co*-MEO$_5$MA)-2, and (C) PS@P(MEO$_2$MA-*co*-MEO$_5$MA)-3 nanogels prepared with same *n*(MEO$_2$MA)/*n*(MEO$_5$MA) feeding molar ratio of 1:3 but different initial monomer (MEO$_2$MA and MEO$_5$MA) amounts of shell in 100 mL of 0.227 g L^{-1} PS dispersion.

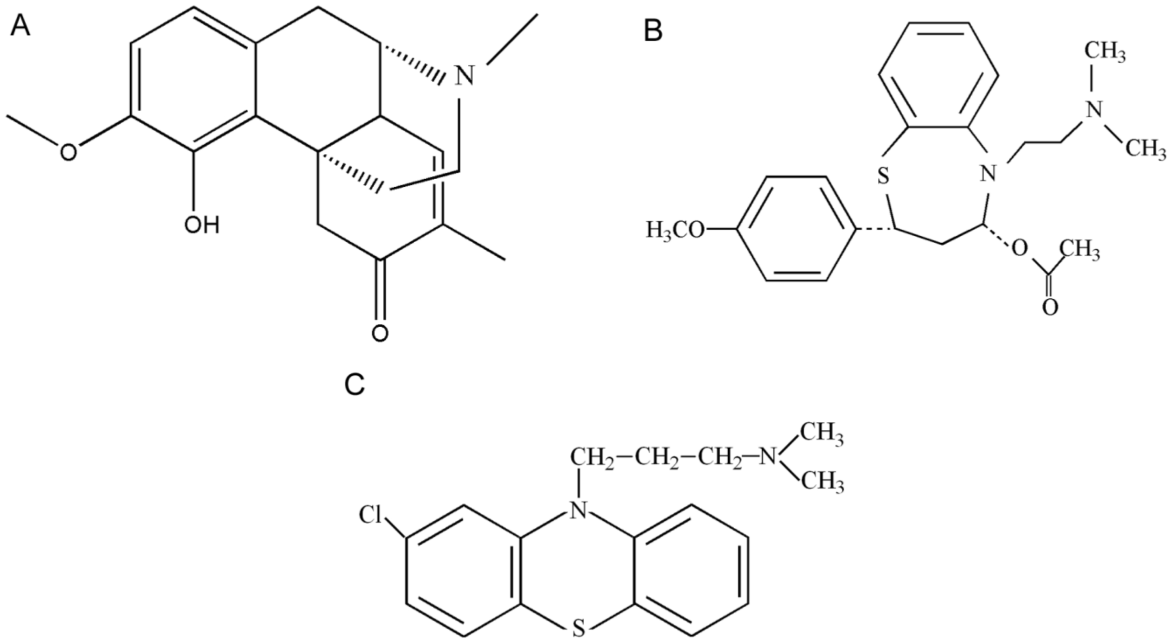

Figure S2. Chemical structures of (A) sinomenine, (B) diltiazem and (C) chlorpromazine.
